# The COVID-19 Pandemic and Explaining Outcomes in Africa: Could Genomic Variation Add to the Debate?

**DOI:** 10.1089/omi.2022.0108

**Published:** 2022-11-14

**Authors:** Nyarai D. Soko, Sipho Dlamini, Mpiko Ntsekhe, Collet Dandara

**Affiliations:** ^1^ Pharmacogenomics and Drug Metabolism Research Group, Division of Human Genetics, Department of Pathology, Faculty of Health Sciences, University of Cape Town, Cape Town, South Africa.; ^2^ UCT/South African Medical Research Council (SAMRC) Platform for Pharmacogenomics Research and Translation, Cape Town, South Africa.; ^3^ Division of Infectious Diseases, Department of Medicine, Groote Schuur Hospital, University of Cape Town, Cape Town, South Africa.; ^4^ Division of Cardiology, Department of Medicine, Groote Schuur Hospital, University of Cape Town, Cape Town, South Africa.

**Keywords:** COVID-19, SARS-CoV-2, genomics, Africa, epidemiology, public health

## Abstract

Severe acute respiratory syndrome coronavirus 2 (SARS-CoV-2), the etiological agent of COVID-19, emanated from the Wuhan Province in China and rapidly spread across the globe causing extensive morbidity and mortality rate, and affecting the global economy and livelihoods. Contrary to early predictions of “body bags” across Africa, the African COVID-19 pandemic was marked by apparent low case numbers and an overall mortality rate when compared with the other geographical regions. Factors used to describe this unexpected pattern included a younger population, a swifter and more effective national health policy, limited testing capacities, and the possibility of inadequate reporting of the cases, among others. However, despite genomics contributing to interindividual variations in many diseases across the world, there are inadequate genomic and multiomics data on COVID-19 in Africa that prevent richer transdisciplinary discussions on the contribution of genomics to the spread of COVID-19 pandemic. To invite future debates on comparative studies of COVID-19 genomics and the pandemic spread around the world regions, this expert review evaluates the reported frequency distribution of genetic variants in candidate genes that are likely to affect COVID-19 infection dynamics/disease outcomes. We propose here that genomic variation should be considered among the many factors determining the COVID-19 infection and its outcomes in African populations and across the world.

## Introduction

The period from December 2019 to the present day in June 2022 has been marked by a global pandemic that started in Wuhan, China (Chen et al, 2020), spreading across the world, leaving a trail of extensive morbidity and mortality rates. COVID-19 is characterized by pneumonia (Zhu et al, 2020) as well as systemic effects in other organ systems. The disease is caused by the severe acute respiratory syndrome coronavirus 2 (SARS-CoV-2) and its various strains (Zhou et al, 2020). The pneumonia was termed COVID-19 (Cucinotta and Vanelli, 2020) by the World Health Organization and subsequently declared a global pandemic in March of 2020 (Cucinotta and Vanelli, 2020). Consequently, as of the end of June 2022, there were a total of 541 million (WHO, 2022a) confirmed cases across the globe and 6.3 million (WHO, 2022a) global deaths since its inception.

## COVID-19 Infection in Africa

**FIG. 1.**
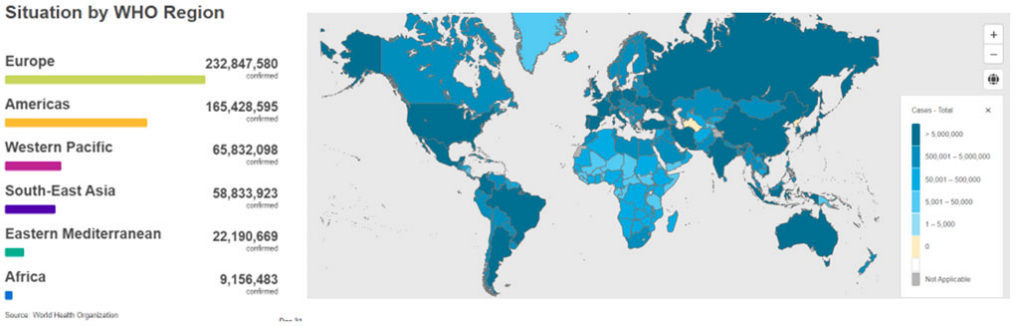
Cumulative global COVID-19 confirmed cases by WHO regions as of June 30, 2022 (adapted from WHO, 2022a).

**FIG. 2.**
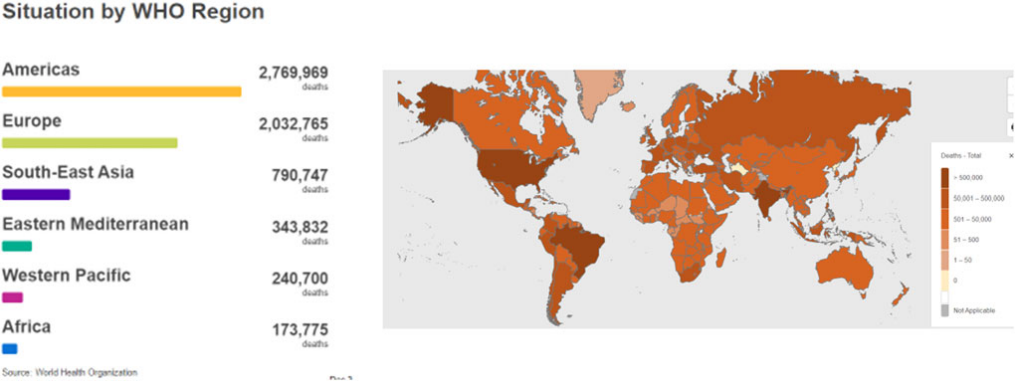
Cumulative global COVID-19 confirmed deaths by WHO regions as of June 30, 2022 (adapted from WHO, 2022a).

COVID-19 infection and mortality rates have distinct geographical patterns (Figs. 1 and 2) marked by high infectivity and an overall mortality rate in the Western hemisphere, and an apparent low overall mortality rate and infectivity in Africa. As of June 2022, there were ∼9 million confirmed cases in Africa and about 172 thousand deaths representing ∼4% of the 541 million cases confirmed globally and 9% of all global deaths to COVID-19. Vaccination played a role in averting at least 19.8 million deaths globally (Watson et al, 2022). Vaccination, therefore, substantially altered the course of the pandemic.

However, low vaccine coverage limited the number of averted deaths in Africa to between 4 and 24 per 10,000 people depending on the country versus 59–170 per 10,000 people observed in high-income countries. Inadequate access to vaccines in low-income countries such as those in Africa meant vaccination had little impact on the pandemic within the years 2020 and 2021 (Watson et al, 2022).

African health care systems have numerous inadequacies ranging from insufficient health infrastructure, limited budgets, to inadequate access to health care staff (Quaresima et al, 2020). The populations are also plagued with a triple burden of infectious endemic diseases such as HIV and tuberculosis (TB), tropical diseases such as schistosomiasis, and noncommunicable diseases such as hypertension. African countries often constitute the bottom of the United Nations Development Program (UNDP) Human Development Index, which measures, among other things, life expectancy, access to quality education, and a decent standard of life. It is, therefore, no surprise that early infection models on morbidity and mortality rates (Lukman et al, 2020; Quaresima et al, 2020) predicted a severe epidemiological picture of COVID-19 in Africa. The United Nations, Economic Commission for Africa in April of 2020 (United Nations ECA, 2020), predicted 300,000 to 3.3 million deaths across Africa during the course of the pandemic.

Contrary to these predictions, cases in Africa remain subdued contributing <4% (WHO, 2022a) to confirmed global cases, the overall mortality rate is lowest in Africa (9% of global deaths) while the World Health Organization (WHO) Africa region (Griffiths et al, 2020) states 80% of reported cases in Africa are asymptomatic (Tessema and Nkengasong, 2021).

Much speculation has been tabled as to the cause of low confirmed cases as well as the lower overall mortality rates of COVID-19 in Africa. In a comprehensive review, Adams et al (2021), post six main hypotheses that attempt to explain why Africa had less COVID-19 disease burden and a lower mortality rate than the other WHO regions. These six trends are as below.

### The demographic structure of Africa may lessen disease burden

The authors' (Adams et al, 2021) arguments are based on trends and common risk factors in regions where COVID-19 infection and mortality rates were high, such as declaration by the CDC that 80% of deaths occurred in patients older than 65 (CDC, 2022) years. Comparing Uganda and Canada, which have similar population numbers, the authors show that the Ugandans have a younger population with a median age of 18 years compared with the 32–42 years for the rest of the world. Africa accounts for 17.2% of the global population yet it has a median age of 18 years (Bamgboye et al, 2021). Only 3% (Quaresima et al, 2020) of the population in Africa is older than 65 years.

It is generally accepted that this young population in Africa aided the asymptomatic nature and low infectivity. Mbow et al (2020) even attribute this younger population to a swifter attainment of herd immunity that led to a lower number of reported cases.

### Lack of long-term care facilities in Africa

Long-term care facilities were cited as centers for the spread of infection and accounted for 81% of deaths to COVID in Canada alone (Webster, 2021), in contrast, the elderly in Africa do not typically reside in long-term care facilities and are often embedded in family structures. In addition many communities in Africa are rural and widely dispersed (Bamgboye et al, 2021) slowing down the spread of the virus. The less dense villages combined with the cultural practice of caring for the elderly at home instead of long-term care facilities imply that the elderly, who are the most vulnerable, are kept in less crowded environments and hence limit the spread of SARS-CoV-2.

### Cross-protection from other circulating coronaviruses or infectious agents

African populations are exposed to a high burden of infectious diseases (i.e., HIV, TB, malaria, and other circulating viruses), which gives potential broad immunity. Heightened exposure to these previous infections is speculated to be a possible reason for a milder presentation of COVID-19 infection (Bamgboye et al, 2021). Speculation also points to previous exposure to similar coronaviruses that may have conferred African populations' immunity.

### Limitations in SARS-CoV-2 testing

Limited SARS-CoV-2 testing in many African countries is also believed to have contributed to potentially undercounting of confirmed infections and deaths. Preliminary seroprevalence surveys show that seroprevalence in high-risk groups ranged from 0.4% (Cape Verde) to as high as 49% (Kenya) (Tessema and Nkengasong, 2021), with the general trend showing higher SARS-CoV-2 seroprevalence than confirmed reported cases. The discrepancies between seroprevalence, which indicates actual infection, and reported cases could be masked by the asymptomatic nature of the COVID-19 in African populations. Asymptomatic cases generally did not seek testing. It is possible that infectivity in African populations was higher than reported, but severe outcomes much lower; this can only be ascertained from population-based seroprevalence studies among African populations living in Africa.

In addition, we note that outcome data are highly sensitive to what is reported by each nation's state administrators, especially in the case of the WHO statistics on COVID-19. Some governments chose not to report, such as Tanzania, while other governments underreported the COVID-19 incidence and prevalence, for various reasons. This may potentially result in skewed or biased global statistics.

### Public health mitigation studies

Previous experience in managing other infectious epidemics such as Ebola and Lassa fever was beneficial to many African countries. Effective national public health response in Africa marked by swift screening programs and travel restrictions both within and across countries is also cited as a potential contribution to reduced infection and mortality rates within the African region. Governments were swift, closing borders and limiting international travel even before cases were reported in their own countries. This greatly limited cases being imported into African countries. In addition, diseases surveillance and contact tracing were rapidly implemented.

### Genetic factors for severe COVID-19

Finally, the authors (Adams et al, 2021) cite genetics as another contributing factor, but do not discuss genetics of COVID-19 in African patients. They argue that environmental factors such as poverty, overcrowding, and working in essential services play a more significant role in ethnic differences across populations than do genetic factors. However, genetic factors that contribute to increased susceptibility, severity of disease, and COVID-19 mortality rates have been reported (Aung et al, 2020; Calabrese et al, 2021; Delanghe et al, 2020; Fawzy et al, 2022; Gintoni et al, 2022; Glotov et al, 2021; Pati et al, 2020), although with few reporting on populations of African descent especially Africans living in Africa.

Questions still arise as to what role did genomics play on the response of individuals living Africa to SARS-CoV-2 infection, which causes COVID-19? Do populations in Africa harbor genomic variants that play a protective role to SARS-CoV-2 infection? If so, to what extent does genetics/genomics contribute to the lower disease burden and mortality rates observed in the African region? This expert review highlights human genome variation and discusses the ways in which genomics might contribute to COVID-19 susceptibility, severity of disease, and mortality rate. This article also invites future comparative genomic studies of COVID-19 infection and its outcomes among Africans and across the world.

## Methodological Approach

A literature search was carried out in PubMed, Google Scholar, and Google. The primary/principal search words were SARS-CoV-2, COVID-19, genomics, genetics, and Africa. The following secondary search words were used to further narrow down the searches, seropositivity, mortality, testing, severity, asymptomatic, contact-tracing, morbidity, mortality, response, pharmacogenomics, and demographic factors (e.g., COVID-19 AND Africa, SARS-Cov-2 AND seropositivity, COVID-19 AND mortality). We were interested in reports that included genomic variation. Although we focused on publications that reported on genomic variation and outcomes of SARS-CoV-2 infection, we understand that in the current historical moment of the pandemic, the main determinants of COVID-19 public health outcomes are social and political, particularly as they pertain to decisions on locking down affected regions, access to vaccines, problems with poor global governance of health, and also uncritical and historically uninformed governance of planetary health (Boschele, 2021).

There are also long-standing social injustices that were laid bare and further deepened (racism, colonialism, class-based discrimination in health care, among others). For example, vaccine access was fraught with hoarding by the rich nations, and the little that became available for the poorest countries, they were priced much more than what richer nations paid.

Focusing on genomic variation, we then teased out all variants that did not show statistically significant differences in frequencies across world populations and excluded them from analysis. Our reasoning was that those similar frequencies would potentially not contribute to the observed interindividual or interpopulation variation. We proceeded to discuss genes with variants that showed variation across populations, for their contribution to the observed differential susceptibility to COVID-19, showing marked interpopulation variation.

### The genomic debate

#### Genomic variation and susceptibility to SARS-CoV-2 infection

Genetics plays a role of varying proportions in both infectivity and mortality rates of communicable disease in human hosts. It has also been shown to contribute to interindividual variation in disease development, progression, and outcome. Indeed, genetics has been reported to play a role in interindividual differences among COVID-19 patients across the globe (Delanghe and Speeckaert, 2022; Glotov et al, 2021; Pati et al, 2020; Teply et al, 2011), but such reports in African populations are scarce. To stimulate an informed debate on the contribution of genetics to infectivity in Africa, we conducted a review of literature and evaluated reported frequency distribution of genetic variants in genes that are known to affect the SARS-CoV-2/COVID infection dynamics/disease outcomes. The aim was to find out if there were any genetic variants that would stand out as the possible determinants of the observed COVID-19 outcomes. We discuss the genes that have evidence of involvement in COVID-19 infection and severity and have variation that resulted in interindividual differences among patients.

##### Angiotensin-converting enzyme 2 and transmembrane protease serine 2

Angiotensin-converting enzyme 2 (ACE2) is the cellular receptor for SARS-CoV-2 (Yan et al, 2020; Yang et al, 2007). ACE2 is an 805 amino acid long type 1 glycoprotein that is distributed in the lungs, heart, renal, and luminal surfaces of the intestinal cells. Its primary physiological role is to counterbalance the activities of its homologue and renin–angiotensin system (RAS) partner, ACE. Together ACE and ACE2 perform a delicate balance that maintains homeostasis and the overall health of the organs in which they are expressed. Lung examination studies (Zhao et al, 2020) show that 83% of pulmonary ACE2 receptors are located within the alveolar epithelial cell type II, where the receptors facilitate corona viral invasion and replication. Infectivity assays in HeLa cells (Zhou et al, 2020) confirm the involvement of ACE2 in SARS-CoV-2 infectivity.

The virion's spike protein interacts with residues Phe486 and Met82 of the ACE2 receptor (Yan et al, 2020), and transmembrane protease serine 2 (TMPRSS2) then cleaves ACE2 between residues 697 and 716 facilitating spike protein-mediated internalization of the virus (Heurich et al, 2014).

Thus, ACE2 levels and polymorphisms independently affect COVID-19 infection (Fawzy et al, 2022; Gintoni et al, 2022; Itoyama et al, 2004; Khayat et al, 2020). In single-cell RNA expression profiling of ACE2 in normal human lungs, Zhao et al (2020) show that ACE2 expression is higher in Asian than in both African American and Caucasian donors. Differences in expression levels of ACE2 result in differences in susceptibility of patients to SARS-CoV-2 infection.

Polymorphisms associated with *ACE2* expression levels and hence differences in COVID-19 susceptibility, severity of disease, and mortality rate are listed in Tables 1 and 2. Only polymorphisms that show marked frequency variation among global populations are listed. Distribution of *ACE2* polymorphisms differs among different ethnic populations (Table 1). Table 1 shows overall higher frequency of alleles associated with ACE2 upregulation and hence susceptibility to infection. Case in point variants rs7563675 (0.41), rs233574 (0.33), rs699 (0.59), rs4830965 (0.38), rs2158082 (0.47), rs5936011 (0.47), and rs4830893 (0.47) all occur at frequencies above 30% in European populations. In fact, only three ACE2 variants associated with increased protein expression have a high frequency in African populations, namely rs424015 (0.54), rs1476524 (0.51), and rs66291109 (0.55).

**Table 1.** Global Distribution of Alleles Associated with COVID-19 Infection Genes Associated with Increased Susceptibility

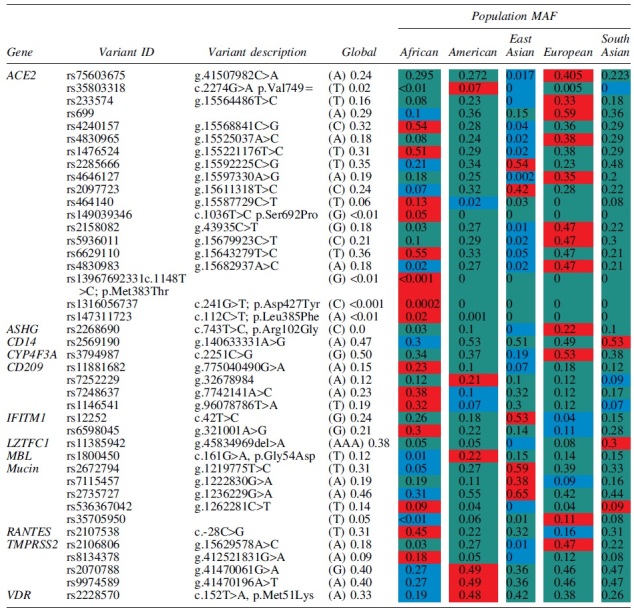

Key: Afr = African; Amr = American; EAS = East Asian; Eur = European; SAS = South Asian; all data extracted from Ensembl Genome Browser Release 106 (April 2022) (Cunningham et al, 2022) unless otherwise stated. Variant code in red highlight = highest population frequency, blue = lowest population frequency while green = middle population frequency.

MAF, minor allele frequency.

**Table 2.** Genes Associated with Decreased Susceptibility and Mortality Rate to COVID-19 Infection

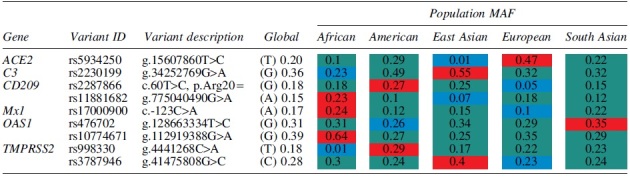

Key: Afr = African; Amr = American; EAS = East Asian; Eur = European; SAS = South Asian; all data extracted from Ensembl Genome Browser Release 106 (April 2022) (45) unless otherwise stated. Variant code in red highlight = highest population frequency, blue = lowest population frequency while green = middle population frequency.

European populations have at least 13 out of the 19 *ACE2* variants listed in Table 1 with a frequency above 20% in their populations versus only 2 in Asian and 3 in African populations. It is possible that the higher expression of ACE2 could have fueled increased susceptibility in European populations when compared with African and Asian populations.

Missense variants rs1396769231 (p.Met383Thr), rs1316056737 (p.Asp427Tyr), and rs147311723 (p.Leu385Phe) occur at higher frequencies in African populations than in both European and Asian populations; these variants have also been associated with the upregulation of *ACE2* expression (Rajeevan et al, 2012). The A allele of rs4240157 occurs at a frequency of 0.17 in the San and as high as 0.67 in the Algerian Mozabite (Osier et al, 2001). This clearly indicates the importance of investigating the genetics of infections such as COVID-19 at the population level, as polymorphisms important in one group may be insignificant in another. Interestingly, the distribution of rs4240157A allele in African populations is high in Algeria, which was among the top African populations infected by SAR-CoV-2 and is fairly common in West Africa (0.44 in the Yoruba) (Rajeevan et al, 2012), while it occurs in lower frequencies in the Bantu (0.25) who constitute the largest ethnic population in Africa.

Africa has a vast heterogenous genetic population, and therefore, genetic contribution in Africa may need to be investigated population by population.

Once viral S protein is bound to the host receptor ACE2, it must undergo fusion to the host cellular membrane to ensure that viral content empties into the host cell. Priming of the viral S protein facilitates fusion of the virus to the host cellular membrane. Proteases such as furin and TMPRSS2 (Bestle et al, 2020; de Araújo et al, 2022) have been shown to be the primary enzymes involved in priming of the S protein. S protein priming is a complex process that is facilitated by the cleavage of the viral S protein at two main sites, the S1/S2 and S′ sites.

Two distinct fragments are produced, S1 that remains attached to the ACE receptor and S2 that harbors the fusion activity and anchors to the cell membrane (Belouzard et al, 2009). Polymorphisms that affect the expression and activity (Table 1) of either furin or TMPRSS2 therefore can be expected to interfere with successful invasion of the virus into host cells and therefore affect susceptibility, severity, and morbidity of COVID-19 infection.

Polymorphisms that increase TMPRSS2 expression have been reported to increase susceptibility to SARS-CoV-2 infection (Asselta et al, 2020; Li et al, 2022; Schönfelder et al, 2021). In a German case–control study (Schönfelder et al, 2021), the frequencies of three variants rs2070788, rs383510, and rs12329760 between cases and controls were investigated. The intronic variant rs2070788 is characterized by a transition from G to A in intron 11; it has been implicated in higher expression levels of TMPRSS2. This polymorphism occurs at its highest frequency in individuals of European descent (0.46), compared with East Asians (0.36) and populations of African descent (0.27). The rs12329760 (c.478G>A; p.Val160Met) polymorphism is found in the scavenger receptor cysteine-rich domain of TMPRSS2.

Individuals with the TT genotype have the highest protease expression levels. This variant, which is associated with increased COVID-19 susceptibility in Indonesian patients (Wulandari et al, 2021), has higher frequency in East Asians (0.36) compared with both Africans (0.24) and Europeans (0.29). However, only rs383510 was significantly associated with increased susceptibility among German cases (Schönfelder et al, 2021) when compared with controls. This confirms that different variants in the same gene can have different clinical implications in different populations.

Hence, populations need to investigate their own ethnic specific variants that may predispose to infection. Interestingly, in this German cohort (Schönfelder et al, 2021), rs383510 only affected susceptibility to infection and not severity of infection or morbidity. The rs383510 single-nucleotide polymorphism (SNP) is in a putative enhancer and the TT genotype is associated with increased expression of the protein. The rs383510T variant has a high frequency in European populations (0.49) when compared with both East Asians (0.36) and Africans (0.33). Another variant associated with overexpression of TMPRSS2 is rs35074065 (Singh et al, 2021) and occurs in only 8% of Africans, while it is more frequent among Americans (27%), Europeans (43%), and South Asians (35%).

The SNP rs8134378, which sits 13Kb upstream from the *TMPRSS2* gene start site, is believed to upregulate TMPRSS2 protein expression (Clinckemalie et al, 2013) and has been linked to increased susceptibility to H1N1 and H7N9 influenza infection (Cheng et al, 2015). This variant is linked to a European haplotype that consists of rs463727, rs34624090, rs55964536, rs734056, rs4290734, rs34783969, rs11702475, rs35899679, and rs35041537. This haplotype is virtually absent among East Asians, while all variants of the haplotype including rs8134378 occur at an average frequency of ≤10% in African populations (Cunningham et al, 2022).

Overall, African populations appear to have lower frequencies of variants that upregulate *TPRMSS2* expression. Thus, African populations present with TMPRSS2 with reduced capacity for invasion of host cells, which therefore leads to lower susceptibility to infection with SARS-CoV-2. It is imperative to further investigate *TMPRSS2* genetic variation in African populations to fully understand the complete picture of its variability and contribution to susceptibility not only to SARS-CoV-2 infection but also to other related viral infections such as H1N1 and H7N9 influenzas.

##### Human leukocyte antigen system

**FIG. 3.**
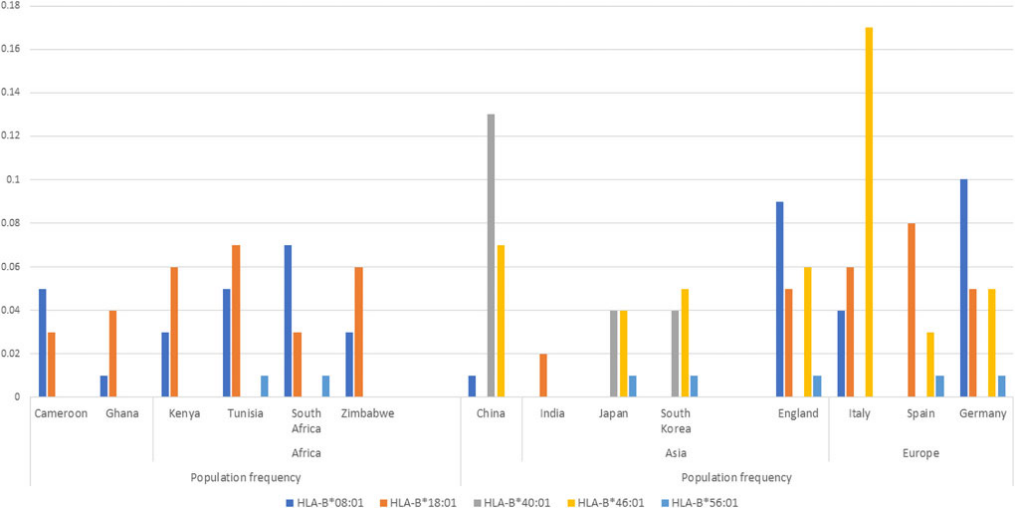
Distribution of HLA-B alleles implicated in COVID-19 response in select populations (allele frequencies obtained from the AFND) (Gonzalez-Galarza et al, 2020). This figure shows a higher frequency of protective allele HLA-B*40:01 in individuals of Asian descent than the other global populations. HLA-B*40:01 seems absent in African populations. HLA-*46:01 is associated with increased risk of COVID-19 infection. HLA-B*46:01 is absent in African populations and is predominantly European.

The human leukocyte antigen system (HLA) encodes components of the major histocompatibility complex and is therefore a vital player in response to viral infection. The risk of SARS infection and severity of the disease, respectively, were reported to be increased among Taiwanese COVID-19 patients harboring HLA-B*46:01 allele (Lin et al, 2003), while in the same Taiwanese patients, the risk of infection was also increased in individuals carrying the HLA-B*54:01 allele. However, a genome-wide association study in COVID-19-infected patients from Spain and Italy (Ellinghaus et al, 2020) reported no significant association between HLA and severity of COVID-19 infection. These HLA-B*46 and *54 alleles are not found in African individuals (Fig. 3), and thus, their effect on COVID-19 infection in African populations is expected to be minimal.

**FIG. 4.**
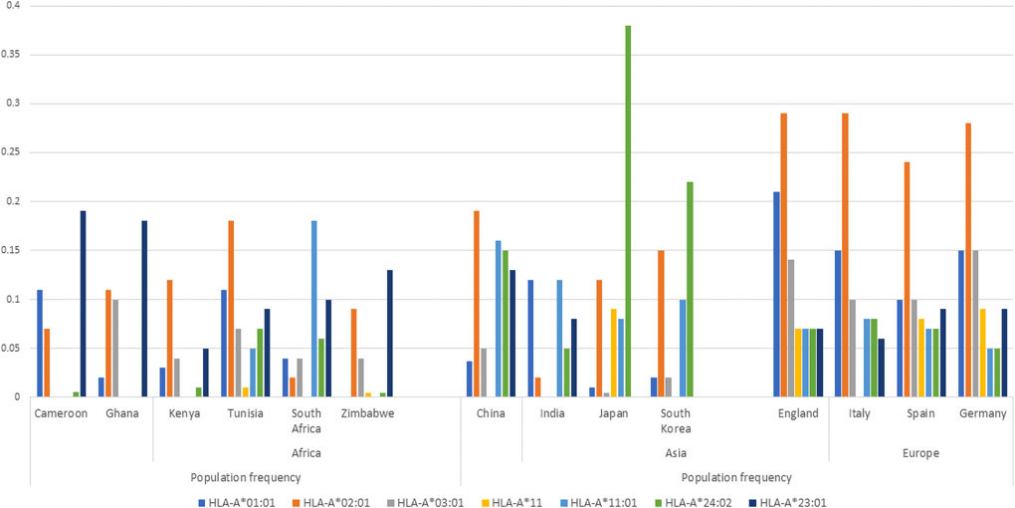
Distribution of HLA-A alleles implicated in COVID-19 response in select populations (allele frequencies obtained from the AFND) (Gonzalez-Galarza et al, 2020). This figure shows a higher frequency of HLA-A*23:01 in individuals of African descent than the other global populations. HLA-*23:01 is reported to have a protective effect on COVID-19 infection. While HLA-A*02:01 (predominantly European) has been associated with low risk of mortality rate in COVID-19 patients. HLA-A*23:01 (predominantly East Asian) is also associated with a protective role against COVID-19 infection. This infers that protective roles may be driven by very different variants among global populations. Interestingly, *HLA-A* alleles associated with increased risk of infection do not vary much among global populations. AFND, allele frequency net database; HLA, human leukocyte antigen system.

HLA-A*01:01 and HLA-B*22 variants have been associated with high risk of SARS-CoV-2 infectivity (Shkurnikov et al, 2021), with the effect of HLA-A*01:01 among African populations likely to be lower than in other global populations as HLA-A*01:01 is found in lower frequencies or rare in Africans (Fig. 4).

##### Chemokines

Chemokines play a vital role in immune response to viral infections and are therefore a deserving target of host genetic investigations into SARS-CoV-2 infection response variations among individuals. One such chemokine implicated in interindividual susceptibility to COVID-19 infection is mannose binding lectin (MBL). MBL activates the selectin pathway of complement and is thus involved in innate immune response to infection. Low levels of MBL affect effective immune response to viral infections. MBL rs1800450 (c.1614G>A, p.Gly54Asp), which has been reported to lower circulating MBL plasma levels and therefore significantly associated with increased susceptibility to SARS-CoV-2 infection (Zhang et al, 2005), occurs in 36% of Chinese patients (Zhang et al, 2005).

The A allele is rarer among African populations (Table 1) occurring at a frequency of 0.01 compared with frequencies >0.10 in other global populations. For example, among Zimbabweans in southern Africa, the rs1800450A variant allele was not found (Mhandire et al, 2014). Chemokine regulated upon activation, normal T cell-expressed and seated (RANTES) has been associated with both susceptibility and severity in Chinese SARS patients. *RANTES* rs2107538 (c.-28C>G) was found in a significantly higher numbers in patients, −28CG and −28GG were both significantly associated with increased susceptibility to SARS infection (Ng et al, 2007). Individuals carrying the −28CG and −28GG genotype, respectively, were reported to have had 2.12-fold and 4.01-fold increased death, respectively, due to SARS-CoV infection. Thus, *RANTES* rs2107538G allele was associated with both susceptibility and death due to SARS-CoV among Chinese patients.

*RANTES* also called *CCL5* is responsible for the recruitment of eosinophils, monocytes, lymphocytes, and basophils to the site of inflammation and thus plays a role in viral infections. Although *RANTES* rs2107538 has not been reported in COVID-19 infection, it does occur at a frequency of 0.45 in African populations (Table 1) when compared with Europeans (0.16) and East Asians (0.32). This particular variant may be of interest to African populations even in an era after COVID-19 as RANTES is an important chemokine in viral response.

##### CD209

*CD209* encodes a protein dendritic cell-specific intracellular adhesion molecule (DC-SIGN) that plays an important role in dendritic response to viral pathogens. Increased expression levels of this protein are linked to increased infectivity of several viral pathogens, including SARS-CoV-2 (Iyer et al, 2020). Three polymorphisms (Table 1), rs11881682, rs7248637, and rs1146541, linked with increased expression are predominant in African populations. rs7248637 occurs at a frequency of 0.92 in the San populations and may be a variant of interest in viral and SARS-CoV-2 infectivity in this population.

##### African variants implicated in a protective role

Among the variants associated with both low infectivity and low mortality rates are variants that are more frequent in African populations. HLA-A*23:1 has been linked to a protective effect, showing a gradient in its frequency across Africa, with higher frequencies in West Africans (20%) (Fig. 4), dropping to around 10% among southern African populations. Again, genetics of SARS-CoV-2 may require more localized geographic investigation in African populations. HLA-A*23 occurs in much lower frequencies in Asian and European populations (<0.05) (Gonzalez-Galarza et al, 2020). It is possible that the protective effect of HLA-A*23 may be more pronounced in West African populations.

**FIG. 5.**
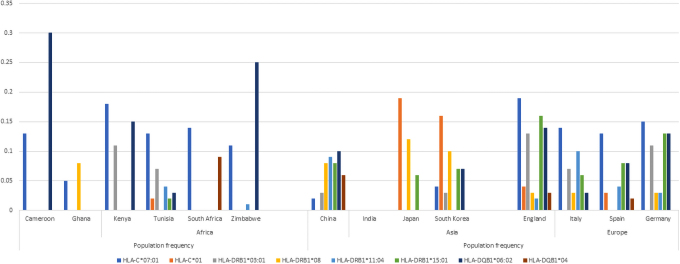
Distribution of various HLA alleles implicated in COVID-19 response in select populations (allele frequencies obtained from the AFND) (Gonzalez-Galarza et al, 2020). This figure shows a higher frequency of allele HLA-DQB1*06:021 in individuals of African descent than the other global populations. HLA-DQB1*06:021 has been associated with an extremely severe course (Iyer et al, 2020) of COVID-19 infection. HLA-C*01 seems absent in African populations, but is present in Japanese and Korean populations; this allele is also associated with a high COVID-19 mortality rate (Iyer et al, 2020).

On the contrary, HLA-A*24:02 and HLA-A*11:01 (Fig. 4) occur at a higher frequency in Asians when compared with African populations. These alleles have been reported to have a protective role on infections (Pretti et al, 2020). Their protective effect therefore is more pronounced in Asian populations than Africans. Alleles HLA-A*02:01 and HLA-A*03:01 were associated with a low risk of mortality rate in Russian COVID-19 patients (Shkurnikov et al, 2021). Both alleles occur at higher frequencies in European populations (Fig. 5). Protective single nucleotide variants (SNVs), *CD209* rs1188168 and *Mx1* rs 1700090, have higher frequencies in African populations when compared with the other global populations (Table 1).

#### Genetics and disease severity

The proportion of deaths due to confirmed COVID-19 infection per region as of end of June 2022 was in Europe (0.9%), Americas (1.73%), Western Pacific (0.38%), Southeast Asia (1.36%), Eastern Mediterranean (1.57%), and Africa (1.91%), against a global average of 1.19%. Despite the low infectivity and low overall mortality rates (Fig. 2) in the WHO African region, the proportion of mortality rate remains highest in the African populations. Indeed, in hospital, mortality rates following initial admission for COVID-19 in Africa were 48.2% (ACCCOS Investigators, 2021) against a global average of 31.5%. In a multicenter, prospective, observational cohort study across 64 African hospitals (ACCCOS Investigators, 2021), the increased mortality rate was attributed to insufficient critical care resources and high prevalence of comorbidities in COVID-19 patients admitted into critical care units. This again speaks of global disparities in resources for health care.

The most common comorbidities in these COVID-19 patients in critical care were hypertension (50.6%) and diabetes (38%); thus, consequently of all the patients who died from COVID-19, 53.8% had hypertension while 42.2% had diabetes (ACCCOS Investigators, 2021). HIV/AIDS, which occurred in 7.7% of the critical care COVID-19 patients, was significantly associated with mortality rate similar to both hypertension and diabetes.

##### Angiotensin-converting enzyme

Levels of both ACE and ACE2, independently, have been associated with susceptibility, severity, and mortality rate of COVID-19 infections (Gintoni et al, 2022; Khayat et al, 2020; Pati et al, 2020). Classical RAS is a pathway that regulates the cardiovascular system through a complex interplay of angiotensin mediators and their receptors. RAS also plays an important role in maintaining the balance of lung cell proliferation or apoptosis (Tan et al, 2018), thus mediating intrapulmonary blood pressure, inflammation, and fibrosis. Dysregulation of RAS has therefore been implicated in pulmonary diseases (Gintoni et al, 2022; Tan et al, 2018) such as chronic obstructive pulmonary disease, asthma, acute lung injury, acute respiratory syndrome, and recently, COVID-19 infection (Calabrese et al, 2021; Khayat et al, 2020; Yan et al, 2020). Dysregulation of RAS is also pivotal in the development of hypertension (Hussain and Awan, 2018).

Increased ACE levels lead to increased production of angiotensin II (AngII), which consequently affects vasoendothelial health and predisposes to cardiovascular diseases such as hypertension, coronary artery diseases, as well as diabetes, renal, and pulmonary disorders all which increase susceptibility to severe and even fatal COVID-19 infection. Expression levels of ACE are linked to a 287 bp insertion/deletion (I/D) mutation in the *Alu* repeat region of intron 16 (Villard and Soubrier, 1996). The ACE I allele results in the presence of the *Alu* repeat, and hence, alternative splicing yields a shorter peptide with one less active site (Purwaningroom et al, 2015).

However, the deletion of *Alu* repeats (D allele) yields enzymes with two active sites and hence increased enzyme activity and production of AngII. Individuals carrying the D allele therefore have higher AngII levels and consequently are at a higher risk of developing AngII-associated pathologies such as hypertension (Sakuma et al, 2004) and diabetes (Kennon et al, 1999; Sakuma et al, 2004). As individuals with the D allele have a higher predisposition to comorbidities that lead to severe COVID-19 infection, and high levels of ACE activity are therefore associated with increased severity and mortality rate of COVID-19 infection (Aung et al, 2020; Calabrese et al, 2021; Delanghe et al, 2020; Pati et al, 2020).

Distribution of ACE I/D polymorphism differs among ethnic groups (Table 1). In a British study (Sagnella et al, 1999), designed to investigate frequency of ACE I/D polymorphism among hypertension and insulin-resistant participants in South London; there was significant difference in frequency of the I allele among the Indian participants (0.40) (*p* < 0.001) when compared with the white (0.18) and African (0.18) groups. However, frequency of the D allele among European (0.32) and African (0.31) patients was not different. Interestingly, within the African group, there was a tendency of higher prevalence of the D allele among hypertensive participants; hypertensive African women had a significantly higher D allele frequency (*p* = 0.003, odds ratio [OR] = 2.54; 95% confidence interval [CI] 1.38–4.65; interaction *p* = 0.023) when compared with their Asian and white counterparts.

Speculation is rife (Calabrese et al, 2021) that this higher incidence of hypertension observed in Africans with the D allele could account for a more severe and fatal COVID-19 infection among individuals of African descent. The idea that the D allele predisposes to susceptibility, severity, and mortality rate of COVID-19 infection is further strengthened by the higher frequency of the ACE D allele in Italians, Spanish, and French Europeans when compared with the Asian ethnic groups (Gemmati and Tisato, 2020).

In contradiction to these findings of the British study (Sagnella et al, 1999), African Americans (Zheng and Cao, 2020) have a higher frequency of the D allele compared with both the Indian and European populations. Similarly, a worldwide spatial study of ACE D allele frequency (Li et al, 2011) showed that Africans and Arabs have the highest global frequencies of the D allele, while East Asians had the lowest frequencies. Hence, the high ACE activities in African individuals could possibly have resulted in a more severe and fatal COVID-19 infection. In addition, low levels of ACE2 are reported in prehypertensive, diabetic, and renal patients of African descent. Mice knockout studies show that reduced expression of ACE2 upregulates ACE/AngII activities, leading to worse lung function characterized by increased vascular permeability, neutrophil accumulation, as well as lung edema, all conditions that increase the severity of COVID-19 infection.

Dysregulation of the delicate balance of ACE and ACE2 in African patients especially those with the DD genotype could possibly have contributed to an increased severity and mortality rate of individuals of African descent infected with SARS-CoV-2. Thus, it is possible that polymorphisms in ACE2 among African populations may have minimal effect on COVID-19 susceptibility, severity of disease, and mortality rate, when compared with the activity of ACE D/I polymorphisms. It is possible that ACE levels and not ACE2, significantly predispose individuals of African descent to a more severe form of COVID-19 owing to predisposition to comorbidities such as hypertension and diabetes. The actual effect of RAS in COVID response among African patients in Africa may shed light on the role of genetics in the severity of SARS-CoV-2 infection in these populations. It potentially can also point to the effectiveness of ACE and ACE2 as potential therapeutic targets among COVID-19 patients.

##### Complement factor 3, CD209, C-C chemokine receptor 5, LZFTL1, and ABO

Genes of the immune system are also implicated in disease severity and increased COVID-19 mortality rate. Complement factor 3 (C3) plays a central role in the immune system and is believed to be pivotal in effective clearance of leukocytes that bind to the vascular wall in COVID-19 patients (Delanghe et al, 2020). Complement gene *C3* is defined by two alleles, slow (S allele) and fast (F allele). The C3 S allele arises due to rs2230199 Asp102 allele. C3 S allele was a significant determinant for COVID-19 mortality rate (*r*^2^ = 0.48, *p* < 0.001). The *C3* S allele occurs at a higher frequency in African populations (Table 1) when compared with other global populations. Similarly, *CD209* rs11881682 occurs in higher frequencies in African populations (Table 1) and has been implicated in increased risk of developing symptomatic COVID-19 (Iyer et al, 2020).

A significant positive correlation between COVID-19 infection rate/million (Spearman *r* = 0.4628, *p* < 0.0001, *n* = 107) and mortality rate/million inhabitants (Spearman *r* = 0.5517, *p* < 0.001, *n* = 107) with frequency of CCRdel32 allele has also been reported (Panda et al, 2020). C-C chemokine receptor 5 (CCR5) is an essential member of the G protein-coupled receptor family involved in the induction of inflammation in viral infections. A 32 bp deletion produces a truncated protein that diminishes surface expression of the receptor. CCrsdel32 has been suggested as an important genetic marker of SARS-CoV-2-related death (Panda et al, 2020). CCR5del32 also showed a positive correlation (Spearman *r* = 0.6210, *p* = 0.0045) with mortality rate in African populations (Panda et al, 2020), however, CCR3del32 is rare in African populations (Solloch et al, 2017), and hence, its effect is expected to be minimal at a population level.

Two loci were reported (Ellinghaus et al, 2020) that are significantly associated with severity of SARS-CoV-2 infection, and further analysis showed that rs11385942 (del>A, g.45834969) in *leucine zipper transcription factor lite 1* (*LZFTL1*) GA allele was more common in patients receiving mechanical ventilation and was associated with increased COVID-19 infection severity (OR for GA allele; 1.77; 95% CI: 1.48–2.11; *p* = 1.15 × 10^−10^). The second locus led to *ABO* rs657152 (g.133263862 A/C/T), which showed a strong association with disease severity (OR for A allele; 1.32; 95% CI; 1.20–1.47, *p* = 4.95 × 10^−8^). The rs657152 A allele occurs at a frequency of 0.44 in African individuals (Table 1), while rs11385942 A insertion is less frequent, occurring in only 5% of African individuals.

##### Genetic variation resulting in inborn errors

Risk factors for severe COVID-19 include the elderly and individuals with comorbidities, when patients do not meet this profile, clinicians and researchers often point to inborn errors or inherited faulty genes. Inborn errors have indeed been associated with young patients (van der Made et al, 2020), typically younger than 40 who present with severe COVID-19 infection. Using whole-exome sequencing, Dutch investigators show the role of an inborn error in *Toll-like receptor 7* (*TLR7*) in young Dutch men with severe COVID-19 infection aged younger than 35. All patients were admitted into intensive care and required mechanical ventilation. They identified a 4-nucleotide hemizygous deletion (c.2129–2132 del), p. [Gln710Argfs*18) in two of the patients who were brothers, and one of them passed away.

This loss-of-function deletion resulted in suppressed TLR7 function and consequently impaired upregulation of interferon genes *IRFZ*, *IFNB1*, and *ISG15* as well as abrogated production if type II IFN and IFN-γ. The second patient had a missense deleterious variant c.2383G>T, p[Val795Phe].

Loss-of-function variants in *TLR7* result in poor identification of single-stranded RNA (ssRNA) in viral infection including infection with SARS-CoV-2 (Bortolotti et al, 2021) and thus possibly allow development of severe COVID-19 infection through reduced ability of the innate immune system to identify viral infection. Another group of inborn errors include autosomal deficiencies in *IFNARI* (*IFR7*) (Zhang et al, 2020). Monogenic inborn errors of type 1 interferons, although rare, have been reported in four adult patients with critical COVID-19 pneumonia (Abolhassani et al, 2022; Zhang et al, 2020). Autoantibodies against type 1 interferons account for at least 10% (Zhang et al, 2020) of critical COVID-19 cases.

Although inborn errors are rare, the possibility of inborn errors and disease severity in African populations needs to be investigated. This is especially so if we consider the absence of genetic investigation in most African cases driven predominantly by the limited output of genetic diagnosis and research in African populations.

##### Y-chromosome DNA haplogroups

Genetic variation in the Y-chromosome influences immune inflammatory response to influenza A (Krementsov et al, 2017) as well as susceptibility to COVID-19 infection (Delanghe et al, 2020). Y-DNA haplogroups are defined by mutations in the nonrecombining portions of DNA from the male Y chromosome. Genetic variation of geographic regions and continents has been explained previously using Y-DNA haplogroups (Woods et al, 2005). A remarkable correlation with COVID-19 susceptibility and the R1b-S116 haplotype among Italians in the Bergamo region has been reported (Delanghe et al, 2020).

Among Africans living in sub-Saharan Africa, the E haplogroup remains the predominant Y-chromosome haplogroup (Woods et al, 2005). The R haplogroup occurs in 5.2% of the African population (Woods et al, 2005) and has a high frequency in northern Cameroon at 95.5% (Cruciani et al, 2002), Sudan (Hassan et al, 2008), Chad (Cruciani et al, 2010), and in Caucasians living in South Africa (Naidoo et al, 2010) where it occurs at a frequency of 50%. The effect of Y-R haplogroup on COVID-19 susceptibility, across the African continent, can be expected to be minimal except in populations where this haplogroup is present in high frequencies.

### In conclusion, should genetics be included in COVID-19 in Africa debate?

COVID-19 is a viral communicable disease, predominantly of the respiratory tract. The causative infectious agent is the coronavirus SARS-CoV-2. Similar to most infections, various factors play a role in the spread of the disease, susceptibility of individuals, severity of disease outcomes, and the general cause of the pandemic. The COVID-19 pandemic in Africa has been driven by numerous factors. Africa has a young population; COVID-19 in other populations affected mostly the elderly in care institutions. Care facilities are not common in Africa. Furthermore, underreporting due to low testing capacity may also add to the biased statistics.

Seroprevalence studies show a larger infection rate when compared with confirmed reported cases. Infection involves interaction between the host and the pathogen. Interindividual variability in susceptibility and severity of infection are both factors of host–pathogen interaction (Frodsham and Hill, 2004; Kwok et al, 2021; Patarcic et al, 2005). Based on this premise, genetics of the African host cannot be sidelined when considering the COVID-19 pandemic in the African population. The benefits of understanding the role of genomics in COVID-19 in Africa spread beyond just aiding explanation of the course of the pandemic, they also offer potential insights into pathogenesis, potential drug targets, risk stratification, and response to therapy and vaccination (Kwok et al, 2021).

It is useful, therefore, to discuss the role of genomics in relation to COVID-19 as well as in response to current and future therapeutics, and hence, this article aims to stir an interest in the role of genomics in COVID-19 pandemic in Africa.

Overall, the COVID-19 infection in Africa is predominantly asymptomatic; hence the low numbers of reported and confirmed cases when compared with the global average. The underlying genetic variation among global populations cannot be ignored and has been shown to play a role in varying infectivity of viral infections including COVID-19 infection (Delanghe and Speeckaert, 2022; Glotov et al, 2021). The African response to COVID-19 infection is heterogenous as is the African population genetic heterogeneity. The full picture of the effect of interethnic and interindividual genetic variation on COVID-19 in African populations therefore needs to be investigated to give a holistic view of the level of infectivity of SARS-CoV-2 in African populations. Varying effects of different gene variants and expressions in other global populations strengthen the call for including genetic variation in unraveling the puzzle of the course of COVID-19 infection in the African continent.

Risk factors associated with severity of COVID-19 infection include an age older than 65 years, male gender, comorbidities such as hypertension and diabetes, as well as obesity. The specific contribution of underlying genetic factors to COVID-19 infection is still to be ascertained, however, emerging studies already point to interindividual differences in similar populations in the same geographical regions owing to interindividual genetic variation (Delanghe and Speeckaert, 2022; Glotov et al, 2021; Zhang et al, 2022). It can therefore be expected that the underlying genetic variation in African populations residing in Africa could play a role in the imprint of COVID-19 infection in the region.

Infection in Africa cannot be confidently based on reported and confirmed cases. Africa has a predominantly young population with a median age of 19 years, and only 3.5% of its population is older than 65 years, hence infection was predominantly asymptomatic as indicated by emerging seroprevalence studies. Emerging seroprevalence studies point to a higher infectivity and strengthen the asymptomatic nature of COVID-19 infection in Africa. Investigation into the underlying genomic variation in asymptomatic versus symptomatic cases in Africa can potentially yield more understanding into the pathology of COVID-19 in African populations.

Looking at common variants implicated in interindividual susceptibility, a distinct pattern between Africans and other global populations does not emerge. However, it is evident that the European populations harbor a higher frequency of ACE2 variants associated with COVID-19 infection when compared with the African populations. Nevertheless, interindividual differences in patients are present and should be investigated in African populations. Africa is made of heterogenous individuals living in different socioeconomic environments, and hence, investigating genomic drivers of susceptibility for each population should still be a priority, as drivers may differ per location and per population.

When we analyze the variants implicated in severe COVID-19 infection and mortality rate, a distinct footprint begins to emerge. Africa harbors high presence of the D allele of ACE, which has been associated with increased hypertension. Hypertension is an established risk factor in severe COVID-19 infection. The prevalence of hypertension in Africa is estimated at 27%, the highest in the world (WHO, 2022b). It is possible that hypertension could be a driving severity in African patients infected with COVID-19. Genetic predisposition to hypertension among African patients would indeed increase severity of infection. Thus, genetics may play a more significant role when African patients get infected with COVID-19.

The African COVID-19 pandemic is multifaceted, and no one factor can solely contribute to the pattern of the disease. The asymptomatic nature of the disease coupled with low testing uptake and capability result in under estimation of the confirmed cases within African populations. The young median age of Africans, swift intervention of national public health efforts, a largely rural population, as well as prior exposure to various infections, create a population that displays low infectivity to SARS-CoV-2 infection.

However, genetic factors cannot be ignored. Genetic factors have already shown distinct roles in interindividual variation in other global populations, and hence, they can be expected to play a role in African populations. African populations appear to harbor lower frequencies of both ACE2 and TMPRSS2 variants associated with high infectivity, yet comorbidities driven to some extent by genetic factors such as ACE2 D allele contribute to a more severe form of disease compounded by weaker health delivery systems. In our opinion, genomics should be considered in the debate of COVID-19 infection within African populations. Genomic research should be thrown in the mix when investigating the African response to COVID-19 infection and indeed to future infections.

## Abbreviations Used

ACE: angiotensin-converting enzyme
AFND: allele frequency net database
AngII: angiotensin II
C3: complement factor 3
CCR5: C-C chemokine receptor 5
CI: confidence interval
HLA: human leukocyte antigen system
I/D: insertion/deletion
MAF: minor allele frequency
MBL: mannose binding lectin
OR: odds ratio
RANTES: regulated upon activation, normal T cell-expressed and seated
RAS: renin–angiotensin system
SARS-CoV-2: severe acute respiratory syndrome coronavirus 2
SNP: single-nucleotide polymorphism
TB: tuberculosis
TLR7: Toll-like receptor 7
TMPRSS2: transmembrane protease serine 2
